# A Low-Cost, Portable Knot-Tying Trainer for Preclinical Surgical Skills Practice

**DOI:** 10.7759/cureus.107287

**Published:** 2026-04-18

**Authors:** Autumn A Stevens, Tyler DePriest, Rita Bliesner, Annah M Smelley, Kaitlyn Haverty, James R Nolin

**Affiliations:** 1 Medicine, Alabama College of Osteopathic Medicine, Dothan, USA; 2 Mechanical Engineering, University of South Florida, Tampa, USA; 3 Simulation, Alabama College of Osteopathic Medicine, Dothan, USA

**Keywords:** low-cost training device, medical education, surgical simulation, surgical skills training, suturing skills

## Abstract

Background

Preclinical medical students often require additional opportunities for repeated practice to develop familiarity and confidence with foundational surgical skills before clinical clerkships. Access to affordable, portable practice tools outside of formal skills laboratories remains limited. We developed SutureStand, a low-cost, portable knot-tying trainer designed to support frequent, self-directed practice among preclinical medical students.

Approach

We conducted a pilot feasibility study to evaluate the usability and perceived educational value of the trainer among first- and second-year medical students at the Alabama College of Osteopathic Medicine. Participants assembled the device and practiced surgical hand ties during a structured skills session, followed by a survey to assess device perception.

Evaluation

Participants completed an anonymous survey assessing the ease of assembly, comparison with a standard knot-tying board, and the likelihood of future use of the device. Survey responses were analyzed using descriptive statistics, including mode analysis and chi-square testing to explore differences by class year. Participants rated the trainer as comparable to standard knot-tying boards and identified portability, affordability, and size as primary advantages. Most respondents reported high usability and perceived educational value, and a majority expressed interest in continued use of the trainer for future self-directed practice.

Implications

This low-cost, portable knot-tying trainer represents a feasible educational innovation to support early, self-directed surgical skills practice in preclinical medical education. By reducing barriers to access and facilitating repeated practice, the trainer may enhance early engagement with foundational surgical skills. Further evaluation using objective performance measures is warranted to better characterize its educational impact.

## Introduction

Student interest in surgical careers has declined in recent years despite a growing and aging U.S. population, with projections estimating a substantial future shortage of practicing surgeons [1]. Although many factors influence medical students’ career discernment, commonly cited deterrents include limited early exposure to surgical practice and low confidence entering the operating room environment [2,3]. These concerns highlight the importance of early educational experiences that foster familiarity and comfort with foundational surgical skills.

Early acquisition of suturing and knot-tying skills represents a fundamental component of surgical education. Preclinical exposure to these skills has been associated with improvement in student confidence and preparedness during surgical clerkships [4,5]. Although structured instruction is often incorporated into preclinical curricula, many students require additional opportunities for repeated practice to develop technical familiarity and procedural comfort [6,7]. Previous efforts to increase preclinical exposure to surgical education include participation in surgical interest groups [8,9] and implementation of targeted courses or programs aimed at early skills development [10-12]. Both methods are effective in promoting early surgical education; however, structured surgical skills courses may require dedicated instructional resources, and both approaches may have variable capacity to promote self-directed learning. 

Self-directed practice as an adjunct to formal instruction has the potential to support early procedural learning by increasing opportunities for repetition and skills reinforcement [13]. However, access to practice tools outside of formal skills laboratories remains inconsistent. Many commercially available suturing and knot-tying trainers are costly, difficult to transport, or impractical for frequent independent use, potentially limiting opportunities for repeated practice. Such barriers may disproportionately affect students with limited access to institutional resources or dedicated practice spaces.

To address this educational gap, we developed a low-cost, portable knot-tying skills trainer intended to support frequent, self-directed practice among preclinical medical students. The objective of this pilot study was to evaluate the feasibility and potential educational value of the trainer following a structured surgical skills session. Additionally, we sought to assess students' perceptions of the trainer relative to standard practice tools and its potential role in supporting preclinical surgical skills education.

## Materials and methods

Study design and oversight

**Figure 1:**
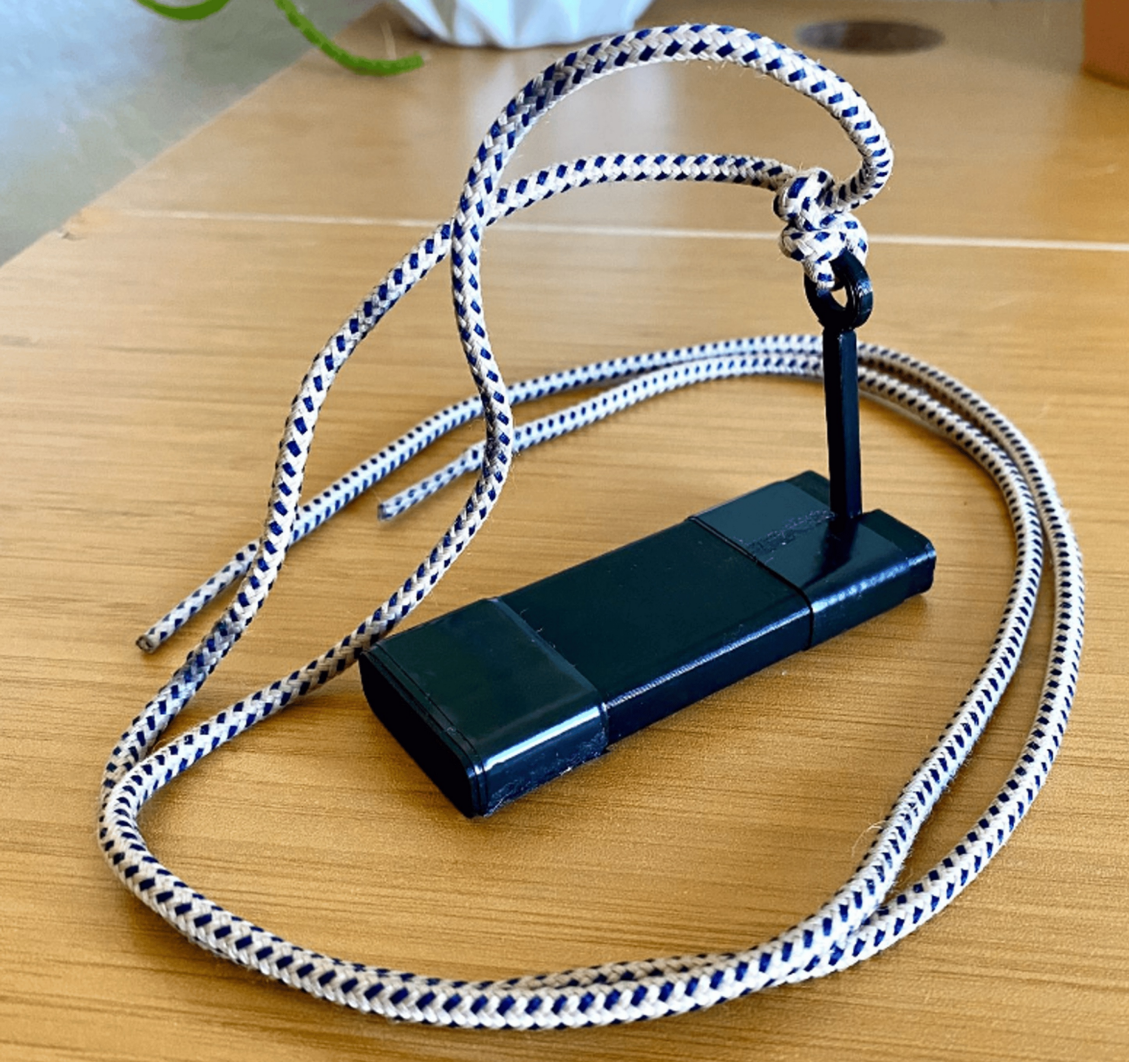
Assembled SutureStand trainer. The trainer consists of three printed pieces and an electrical tape wrapped around each end to provide extra grip and stability.

This pilot feasibility study was approved by the institutional review board at the Alabama College of Osteopathic Medicine, protocol number 24-09-18-001. The study was designed to evaluate the feasibility, usability, and perceived educational value of a low-cost suturing trainer (SutureStand) among preclinical medical students. The device is shown in Figure 1.

Participants and educational setting

First- and second-year medical students enrolled at the Alabama College of Osteopathic Medicine during the 2024-2025 academic year were invited to participate following attendance at a student-led surgical skills clinic. The clinic consisted of a one-hour instructional session on knot-tying and basic suturing techniques, including hands-on practice using a standard knot-tying board with faculty and senior student facilitation.

The knot-tying device used during the instructional clinic and for device comparison in this study was the Ethicon Knot Tying Board (Ethicon Inc., Somerville, NJ, USA). The device measures 8 inches x 8 inches and includes parallel bands for knot-tying and a hook enclosed in a cup to simulate placing knots into deep cavities. The board was selected for use due to its availability at the institution where this study was conducted, but it has similar features to other knot-tying devices available for purchase at major retailers. 

Following completion of the clinic, all attendees were invited to participate in the study. Nineteen students (8 osteopathic medical students (OMS)-I and 11 OMS-II) consented to participate. Eligibility was limited to first- and second-year medical students; no participants were excluded.

Educational intervention

The educational intervention consisted of independent practice using a low-cost suturing trainer designed to support self-directed skills rehearsal. Participants were provided with brief written instructions for assembly and use of the trainer (Appendix A) and were asked to practice surgical hand ties using the trainer for approximately 10 minutes. The intervention was intended to simulate informal, self-directed practice outside of structured teaching sessions, and no formal instruction was provided during the intervention period.

Description of the educational innovation

The knot-tying trainer (SutureStand) was designed to be portable, inexpensive, and accessible due to its compatibility with standard desktop three-dimensional (3D) printing. Nationwide surveys from 2020 and 2021 report that 30-40% of U.S. university settings include access to 3D printers and that this number continues to rise with the growing popularity and affordability of makerspaces [14,15]. Importantly, the device can be assembled from a small number of simple printed components and can readily be fabricated by institutional facilities, collaborators, or commercial printing services if individuals do not have direct access to 3D printers. Detailed design specifications and assembly instructions are provided in Appendix B.

Outcome measures

Following practice, participants completed an anonymous survey assessing the feasibility and acceptability of the trainer. Survey items evaluated the ease of assembly, perceived usability, comparison to a standard knot-tying board, and willingness to use the trainer for future independent practice (Appendix C). Survey items were developed by the study investigators to align with the objectives of this pilot feasibility study and were not formally validated. However, questions were reviewed by investigators with experience in surgical education to ensure clarity and relevance. 

Data collection and analysis

Survey responses were collected using Qualtrics (Qualtrics LLC; Provo, UT, USA) and analyzed using descriptive statistics. Given the pilot nature of the study and small sample size, analyses were exploratory and focused on summarizing participants' perceptions of usability and acceptability. Comparative analyses by class year were performed using chi-square testing where appropriate, with a significance threshold of 𝛂=0.05. All data were analyzed in Microsoft Excel (Microsoft; Redmond, WA, USA). 

## Results

Participant characteristics

Nineteen preclinical medical students participated in this study, including 8 OMS-I and 11 OMS-II students.

Previous suturing experience

Most participants reported limited previous experience with surgical hand ties, with 57.9% (11/19) indicating “a little” experience before attending the skills clinic. All participants reported having used the standard knot-tying board during the clinic session (19/19). These data are summarized in Table 1.

**Table 1:** Students' experiences with hand ties and perception of SutureStand compared to the standard practice board. Most students reported a little experience with hand ties and felt that SutureStand was about the same or better than the standard practice board.

Students' experiences of performing surgical hand ties		
None	3 of 19	15.8%
A little	11 of 19	57.9%
A moderate amount	5 of 19	26.3%
A lot	0 of 19	0%
A great deal	0 of 19	0%
Compared to the standard practice board, SutureStand is		
Much better	3 of 19	15.8%
Somewhat better	5 of 19	26.3%
About the same	10 of 19	52.6%
Somewhat worse	1 of 19	5.3%
Much worse	0 of 19	0%

Trainer acceptability and perceived utility

**Figure 2:**
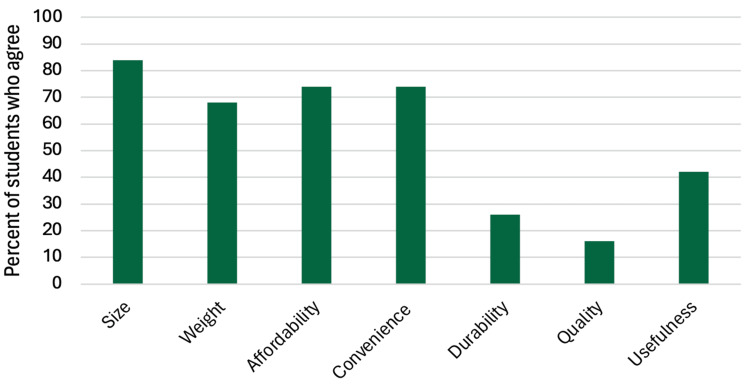
Qualities that make SutureStand superior to the standard practice board. Many students reported that SutureStand had a more favorable size, affordability, convenience, and weight compared to the standard training board. Quality and durability were selected less frequently.

When compared to the standard knot-tying board, 52.6% of participants (10/19) rated the trainer as “about the same.” Attributes most frequently valued by the participants included small size (84.2%, 16/19), affordability (73.7%, 14/19), convenience (73.7%, 14/19), and lightweight design (68.4%, 13/19). Usefulness was selected by 42.1% of participants (8/19), whereas durability (26.3%, 5/19) and overall quality (15.8%, 3/19) were selected less frequently. These findings are illustrated in Figure 2 and summarized in Table 2.

**Table 2:** Qualities that make SutureStand superior to the standard practice board. Many students reported SutureStand’s size, affordability, convenience, and lightweight design to be superior to those of the standard practice board. Quality and durability were less frequently noted to be advantages of SutureStand.

What qualities make SutureStand superior to the standard practice board?		
Size	16 of 19	84.2%
Weight	13 of 19	68.4%
Affordability	14 of 19	73.7%
Convenience	14 of 19	73.7%
Durability	5 of 19	26.3%
Quality	3 of 19	15.8%
Usefulness	8 of 19	42.1%

Exploratory comparisons by class year

Responses from OMS-I and OMS-II students demonstrated similar overall trends. A difference was observed in the perceived ease of assembly, with most OMS-I students reporting the assembly process as “somewhat easy” and most OMS-II students reporting it as “somewhat difficult.” Interest in future use of the trainer was similar across groups, with 63.2% of participants (12/19) indicating they were “somewhat likely” to continue using the trainer if made available through their surgical interest group. These results are summarized in Table 3.

**Table 3:** Difficulty in assembling SutureStand and likelihood of using the trainer in the future. Most students reported the trainer was somewhat or extremely easy to assemble. All students reported being at least somewhat likely to use SutureStand in the future.

Difficulty of assembly		
Extremely difficult	0 of 19	0%
Somewhat difficult	4 of 19	21.1%
Neither easy nor difficult	5 of 19	26.3%
Somewhat easy	7 of 19	36.8%
Extremely easy	3 of 19	15.8%
Likelihood of using SutureStand in the future		
Very likely	7 of 19	36.8%
Somewhat likely	12 of 19	63.2%
Neither likely nor unlikely	0 of 19	0%
Somewhat unlikely	0 of 19	0%
Very unlikely	0 of 19	0%

An exploratory chi-square analysis showed no association between class year and likelihood of future trainer use, χ²(1, N=19)=0.003, p=0.96, Cramér's V=0.01. Similarly, Fisher's exact test demonstrated no significant association (p=1.00). A comparison of observed and expected frequencies revealed minimal deviation, and standardized residuals ranged from -0.03 to 0.03, indicating that no response category was selected significantly more or less often than expected by either group. Given the pilot nature of the study, small sample size, and limited variability in responses, this analysis was interpreted descriptively.

## Discussion

This pilot study evaluated the feasibility and perceived educational value of a low-cost suturing trainer designed to support self-directed practice among preclinical medical students. Our findings suggest that the trainer is acceptable to learners and well-received compared to existing training boards, with notable advantages in portability and affordability that may support more frequent skills practice.

The portability and low cost that the trainer imparts may facilitate greater opportunities for distributed, self-directed practice outside of formal teaching sessions. Previous work has demonstrated that frequent, brief practice opportunities support procedural skill development, especially among novice learners [6,7]. Barriers such as cost, logistical constraints, and restricted access to simulation laboratories can limit opportunities for frequent practice, and reducing these barriers may enable broader participation in surgical skills development across diverse learning contexts.

This trainer may be particularly well-suited for informal and peer-led learning environments, including surgical interest groups and skills workshops. These settings regularly supplement formal curricula but often face constraints related to funding, storage space, and equipment availability. An inexpensive and portable trainer may function as an effective educational adjunct, reinforcing skills introduced in structured teaching sessions and supporting independent practice.

Although the trainer was not perceived as superior to existing knot-tying boards, its acceptability among learners is an important finding. Previous educational literature suggests that access and practice frequency may be more influential for early skill acquisition than high-fidelity simulation tools that are less readily available [16,17]. A trainer perceived as sufficient and accessible may support more consistent engagement and greater learning gains than a potentially superior tool with limited availability.

This pilot study has several limitations. First, a small sample was gathered from a single institution due to the pilot nature of the project, with the intention to assess the feasibility of the design and inform further research. Although limited in generalization, these findings provide encouraging preliminary evidence and support the feasibility and acceptability of the trainer and the need for more extensive product testing. Second, the study was limited in funding and access to additional training devices for comparison to SutureStand. Future studies using a variety of available devices are indicated to strengthen the comparison of the trainer to other surgical education tools. Finally, this study utilized self-reported survey data to assess the perception of the trainer. Self-reported data are susceptible to bias due to their subjective nature and limit conclusions regarding skill acquisition. Therefore, further research utilizing objective measures, such as knot performance, is greatly recommended. 

Future studies should evaluate the impact of this trainer using objective measures of knot-tying performance and skill retention, as well as explore its integration into formal preclinical curricula. Incorporating learner feedback through iterative design refinement may further improve usability and educational value, particularly as the tool is adapted for use in different educational environments.

By lowering barriers to access and supporting frequent, self-directed practice, this educational innovation may enhance early procedural learning opportunities for preclinical medical students beyond the confines of formal skills laboratories. Public availability of design files enables learners and educators to independently fabricate the trainer, further supporting broader access to self-directed preclinical surgical skills practice.

## Conclusions

The authors cautiously conclude that SutureStand represents a feasible and accessible educational tool to support early development of surgical knot-tying skills among preclinical medical students. In this pilot evaluation, students perceived the trainer as comparable to existing practice tools while offering advantages in portability, affordability, and ease of access. By lowering barriers to practice outside of formal skills laboratories, this trainer may facilitate more frequent, self-directed procedural skills during preclinical years. Further refinement of the design and evaluation using objective measures of skill acquisition and a larger sample size are warranted to better understand its impact on surgical education.

## Figures and Tables

**Figure 3:**
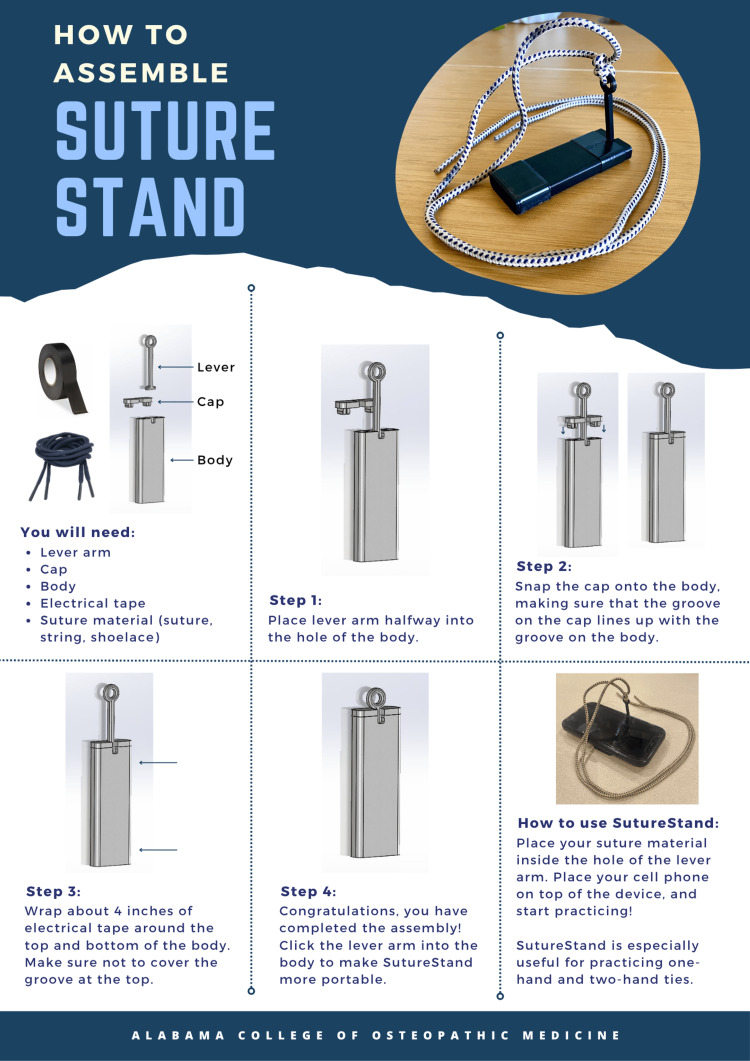
Assembly guide for SutureStand. This infographic describes how to assemble SutureStand in a series of four steps. No artificial intelligence (AI) tool was used to generate this infographic. It was developed by one of the study investigators using Canva (Canva Pyt Ltd., 110 Kippax Street, Surry Hills, NSW 2010, Australia).

**Figure 4:**
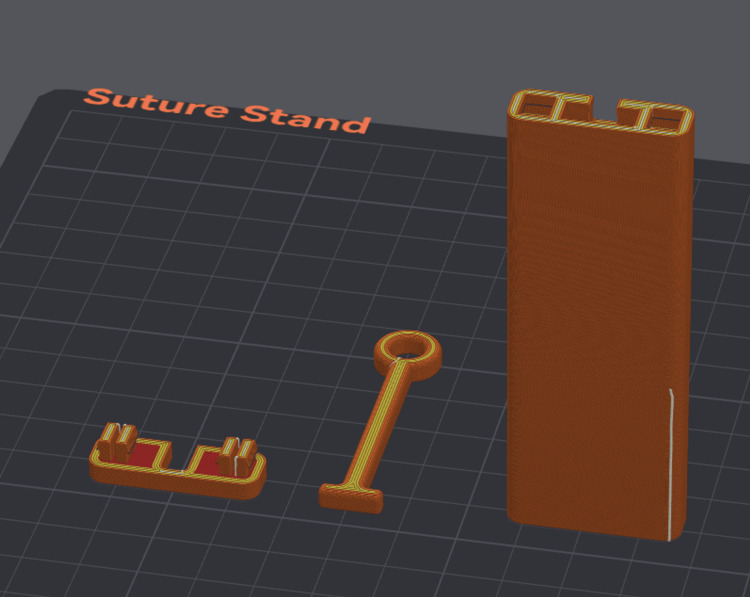
Printing orientation for SutureStand. SutureStand was sliced using Orca Slicer; however, any standard industry slicing software is sufficient to slice the files.

**Figure 5:**
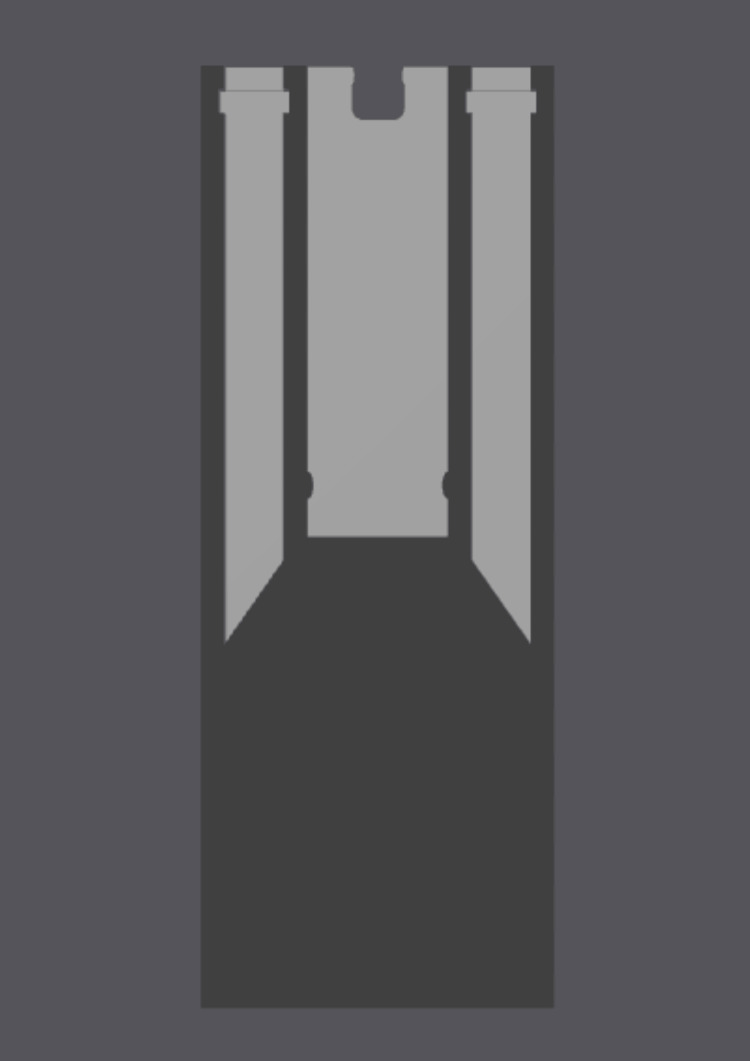
Cross-section of SutureStand body. Design features include a middle channel to store and secure the arm, bilateral detents to affix the cap to the body, and a notch to allow the arm to rotate and lock into place when in use.

## Appendices

Appendix A: assembly guide for SutureStand

Figure 3 shows the assembly guide used by the participants to assemble SutureStand.

Appendix B: SutureStand design and fabrication details

Device Overview

The knot-tying trainer evaluated in this study was named SutureStand to reflect its design and intended function. The device was designed using computer-aided design (CAD) software and fabricated using standard desktop 3D printing techniques. SutureStand was intentionally designed to be low-cost, portable, and printable without support structures to facilitate replication in educational settings.

The design files for SutureStand are publicly available at no cost and may be downloaded for educational use from an online repository (Thingiverse). The files were made available to facilitate replication and self-directed practice by medical students and educators (www.thingiverse.com/thing:6954948).

Materials and Assembly

SutureStand consists of three 3D-printed components: a body, an arm, and a cap. All components were printed using standard polylactic acid (PLA) filament. Devices used in this study were printed using Polymaker PLA; however, any commercially available PLA filament is suitable. For use, additional materials include an electrical tape and a weighted object (e.g., a mobile phone or notebook) placed on top of the device to increase friction and stability during practice.

Fabrication and Print Settings

Devices were printed using commercially available desktop 3D printers. The print settings used for fabrication are summarized in Table 4. Files were sliced using Orca Slicer (https://www.orcaslicer.com/); however, other standard slicing software may be used. The device was designed to print without supports and with minimal overhangs to simplify fabrication. Printing orientation is illustrated in Figure 4.

**Table 4:** Print settings used for the fabrication of SutureStand. Print settings for 3D printing fabrication are described. 3D: three-dimensional

Parameter	Measurement
Layer height	0.2 mm
Infill percentage and pattern	25% minimum, gyroid
Wall layers	3 minimum
Nozzle diameter	0.4 mm
Extrusion temperature	210°C
Bed temperature	60°C
Supports	None

Design Features Relevant to Use

A cross-sectional view of the SutureStand body is shown in Figure 5. Key design features include a central channel to secure the arm during storage, bilateral detents to affix the cap to the body, and a notch that allows the arm to rotate and lock into position during use. Dimensional tolerances were selected to accommodate minor variations across desktop printers while maintaining proper fit and functionality.

Appendix C: survey items

1. What is your class year?

a. OMS-I

b. OMS-II

2. How much experience have you had performing surgical hand ties before today’s workshop?

a. None

b. A little

c. A moderate amount

d. A lot

e. A great deal

3. Did you use the standard knot-tying practice board during today’s workshop?

a. Yes

b. No

4. Compared to the standard practice board, SutureStand is

a. Much better

b. Somewhat better

c. About the same

d. Somewhat worse

e. Much worse

5. What qualities make SutureStand superior to the standard practice board? Please select all that apply.

a. Size

b. Weight

c. Affordability

d. Convenience

e. Durability

f. Quality

g. Usefulness

h. None of the above

6. How difficult was the assembly of SutureStand?

a. Extremely difficult

b. Somewhat difficult

c. Neither easy nor difficult

d. Somewhat easy

e. Extremely easy

7. If SutureStand was provided to you by your surgical interest group, how likely are you to use SutureStand to practice surgical knot ties?

a. Very likely

b. Somewhat likely

c. Neither likely nor unlikely

d. Somewhat unlikely

e. Very unlikely
